# Influence of atrial fibrillation subtypes on anticoagulant therapy in a high-risk older population: the FAI project

**DOI:** 10.1007/s40520-022-02140-w

**Published:** 2022-05-11

**Authors:** Antonio Di Carlo, Fabio Mori, Domenico Consoli, Leonardo Bellino, Augusto Zaninelli, Marzia Baldereschi, Maria Grazia D’Alfonso, Chiara Gradia, Alessandro Cattarinussi, Bruno Sgherzi, Giovanni Pracucci, Benedetta Piccardi, Bianca Maria Polizzi, Domenico Inzitari, for the National Research Program: Progetto FAI. La Fibrillazione Atriale in Italia

**Affiliations:** 1 Institute of Neuroscience, Italian National Research Council, Via Madonna del Piano, 10 – 50019 Sesto Fiorentino, Florence, Italy; 2 Cardiovascular Unit, Careggi University Hospital, Florence, Italy; 3 Azienda Sanitaria Provinciale, Vibo Valentia, Italy; 4 Azienda Unità Sanitaria Locale Toscana Centro, Florence, Italy; 5 The System Academy, Florence, Italy; 6 Department of Neurofarba, University of Florence, Florence, Italy; 7 Agenzia Di Tutela Della Salute, Bergamo, Italy; 8 Stroke Unit, Azienda Ospedaliero-Universitaria Careggi, Florence, Italy; 9 National Centre for Disease Prevention and Control, Italian Ministry of Health, Rome, Italy

**Keywords:** Atrial fibrillation subtypes, Anticoagulants, Thromboembolism, Older population

## Abstract

**Background and aim:**

Benefits of oral anticoagulants (OAC) in atrial fibrillation (AF) patients with moderate-to-high risk of stroke are independent of AF pattern. We evaluated whether AF clinical subtype influenced OAC use in a representative sample of the Italian older population.

**Methods:**

A cross-sectional examination of all subjects aged 65 + years from three general practices in northern, central, and southern Italy started in 2016. A double-screening procedure was followed by clinical and ECG confirmation. Patients were categorized as having paroxysmal, persistent, or permanent AF. OAC use was evaluated in confirmed AF patients.

**Results:**

The sample included 6016 subjects. Excluding 235 non-eligible, participation was 78.3%, which left 4528 participants (mean age 74.5 ± 6.8 years, 47.2% men). Overall, 319 AF cases were identified: 43.0% had paroxysmal, 21.3% persistent, and 35.7% permanent AF. Frequency of OAC therapy was 91.2% in permanent, 85.3% in persistent, and only 43.0% in paroxysmal AF (*P* < 0.001). In multivariate analysis, controlled for baseline variables and risk scales, persistent and permanent AF were associated with a significant increase in the likelihood of receiving OAC compared with paroxysmal AF (P < 0.001). This was confirmed for permanent AF also in multivariate analyses considering separately vitamin K antagonists or direct-acting oral anticoagulants (OR, 4.37, 95% CI, 2.43–7.85; and 1.92, 95% CI, 1.07–3.42, respectively) and for persistent AF and direct-acting oral anticoagulants (OR, 4.33, 95% CI, 2.30–8.15).

**Conclusions:**

In a population-based survey, AF pattern was an independent predictor of OAC treatment. Paroxysmal AF is still perceived as carrying a lower risk of vascular events.

## Introduction

Atrial fibrillation (AF) is the most common cardiac arrhythmia with a clinical relevance, affecting approximately 33.5 million persons worldwide [1]. The prevalence of AF is strictly age-dependent: the overall frequency in the adult population approaches 1–3% [2, 3], exceeding 15% in people aged 80 years and over [3–5], the fastest-growing segment of the older population [6]. Therefore, the number of affected persons and the impact on healthcare costs are predicted to increase dramatically [2, 5, 7].

Despite available treatments, this arrhythmia remains among major causes of cardiovascular morbidity and mortality, including sudden death, stroke, and heart failure [7]. AF increases by four- to five-fold the risk of ischemic stroke [8], accounting for about 20–30% of total events [7]. AF-related stroke is severe, and has a poor prognosis in terms of survival and residual disability [9]. Although oral anticoagulants (OAC) may prevent up to two-thirds of ischemic strokes in AF patients [10], this therapy is still far from an optimal use [11].

Clinical subtypes of AF, identified as paroxysmal, persistent, and permanent forms, and defined according to presentation, duration, and termination of episodes, may influence overall cardiovascular risk and management decisions [7, 12]. In recent trials, persistent and permanent AF significantly increased the risk of stroke, systemic embolism, and premature death compared with paroxysmal AF [12–16]. In secondary stroke prevention, sustained AF was also associated with a significantly worse 3-month functional outcome compared with paroxysmal AF [17]. However, clinical trials clearly indicated also the benefits of OAC in patients with moderate-to-high clinical risk of stroke regardless of AF pattern [13–16].

The aim of this study was to estimate whether AF subtypes influenced the use of OAC therapy in a large representative sample of the Italian older population.

## Methods

### Study design and population

This survey is part of the National Research Program: Progetto FAI. La Fibrillazione Atriale in Italia (FAI Project. Atrial Fibrillation in Italy), funded by the National Centre for Disease Prevention and Control of the Italian Ministry of Health, and coordinated by the Tuscany Region. The project had a prospective multicentre design, including a cross-sectional investigation, which started in March 2016, of all subjects aged 65 years and older from three Italian primary care practice cohorts, and a follow-up assessment of patients diagnosed with AF 6 months after the baseline examination. Enrolment ended in June 2017, and last follow-up examination was performed in December 2017.

The methodology has been described in detail elsewhere [5]. In short, to ensure a national representativeness, the three practices covered urban and rural areas, different socio-economic contexts, and were located in northern (Lombardy, Romano di Lombardia), central (Tuscany, Florence), and southern (Calabria, Vibo Valentia) Italy. A fourth research unit was set in the Cardiothoracic and Vascular Department of Careggi University Hospital in Florence and was in charge of centralized electrocardiograms (ECG) reading and AF diagnosis.

A computerized list of all patients aged 65 years and older was obtained from each practice, to identify the initial study sample. This comprised 2005 subjects from Lombardy, 2009 from Tuscany, and 2002 from Calabria, for a total of 6016 individuals. All these were considered potentially eligible. Exclusion criteria were only those limiting an effective participation: moved to another address, changed the general practitioner (GP), and died between data extraction and starting of the survey, terminal disease, severe dementia, refusal to participate.

To minimize interobserver variability, before the study, all the field personnel had a centralized training focused on research design, study protocol, and questionnaires. The study complies with the Declaration of Helsinki, and the research protocol was approved by the ethics committees of Azienda Ospedaliero-Universitaria Careggi, Florence, Agenzia di Tutela della Salute, Bergamo, and Azienda Sanitaria Provinciale, Vibo Valentia. Written informed consent was signed by all participants according to institutional guidelines.

### Screening procedure

To increase the robustness of prevalence estimates, we used a double-screening procedure to identify AF cases, including a systematic and an opportunistic screening of the study population. Methods, limits, and results of these methodologies have already been reported in detail [5]. Both screening procedures were followed, in positive cases, by a direct diagnostic assessment, in their own primary care practice, to confirm or not the suspect of AF. This included medical history, a clinical examination about possible symptoms of AF, evaluation of radial pulse, detection of pacemaker or implantable cardioverter–defibrillator, and the recording of a 12-lead ECG.

### Diagnostic confirmation

A 12-lead ECG read by a cardiologist is recognized as the gold standard for the diagnosis of AF [18]. For all subjects with a positive screening, a 12-lead ECG at rest was recorded and digitally stored at each practice, using My CardioPad XL ECG Recorder (Esaote, Florence, Italy). Independently of results, to verify the presence of AF, all ECGs were centrally read by one cardiologist (M.G.D.A.), expert in electrophysiology and cardiac arrhythmias, of the Cardiologic Research Unit in Florence. For patients reporting a previous diagnosis of AF, not evidenced by the ECG performed during the survey, this had to be confirmed by the study cardiologist based on ECGs retrieved from the medical records and on previous specialist diagnosis. Information on the date of onset, symptoms, and characteristics of AF was collected. Risk scales CHA_2_DS_2_-VASc [congestive heart failure, hypertension, age ≥ 75 (doubled), diabetes, prior stroke or transient ischemic attack (doubled), vascular disease, age 65–74, female sex] [19] and HAS-BLED [hypertension, abnormal renal/liver function, stroke, bleeding history or predisposition, labile international normalized ratio, elderly (> 65 years), drugs/alcohol concomitantly] [20] were also administered. CHA_2_DS_2_-VASc risk score ≥ 2 was considered a formal indication for anticoagulant therapy in the absence of contraindications, while an HAS-BLED score ≥ 3 indicated a high risk for bleeding. Use of specific therapies was recorded, including antiplatelets, vitamin K antagonists (VKA), three currently available (dabigatran, rivaroxaban, and apixaban) direct-acting oral anticoagulants (DOAC), and pharmacological and electrical cardioversion. All patients with a previous diagnosis of AF confirmed by the study cardiologist were included in the present report.

Medical history and all clinical material collected during the survey were evaluated for the diagnosis of AF subtypes, which was made after the 6-month examination. To increase diagnostic reliability across centres, the subtype attribution was made by an adjudication panel including the cardiologist of the Cardiologic Unit, medical personnel from each research unit, and the methodology group of the Project.

European Society of Cardiology diagnostic criteria were used for AF subtypes [7], collapsing, for the study purposes, long-standing persistent AF into persistent AF. *Paroxysmal AF*: self-terminating, in most cases within 48 h. Some AF paroxysms may continue for up to 7 days. AF episodes that are cardioverted within 7 days should be considered paroxysmal. *Persistent AF*: AF that lasts longer than 7 days, including episodes terminated by cardioversion, either with drugs or by direct current cardioversion, after 7 days or more. Continuous AF lasting for ≥ 1 year when it is decided to adopt a rhythm control strategy. *Permanent AF:* AF that is accepted by the patient (and physician). Hence, rhythm control interventions are, by definition, not pursued in patients with permanent AF.

### Baseline variables

For all participants, data gathering was performed through structured questionnaires from the computerized practice records, from the GPs or directly from the patients. Information was collected on age, sex, living conditions, and education; current therapies; and vascular risk factors and comorbid conditions: hypertension (previous diagnosis, current treatment, or values ≥ 140/90 mmHg in at least 2 subsequent measurements), previous myocardial infarction (diagnostic ECG, documented hospital discharge or diagnosis by a specialist), heart failure (diagnosed by a cardiologist evaluating symptoms, signs, electrocardiography, chest X-ray, and echocardiography), diabetes mellitus (previous diagnosis or on antidiabetic medication), hypercholesterolemia [total cholesterol level ≥ 200 mg/dL (5.18 mmol/L)], hypertriglyceridemia [triglycerides’ level ≥ 150 mg/dL (1.7 mmol/L)], alcohol consumption, peripheral artery disease, renal disease [chronic dialysis, renal transplantation, or serum creatinine ≥ 2.26 mg/dL (200 μmol/L)], previous stroke, and history of transient ischemic attack.

### Statistical analysis

Continuous variables are presented as means and standard deviations, and categorical variables as percentages. The Chi-square test was used to compare categorical variables, and analysis of variance for the continuous variables. Logistic regression analysis, including demographics, vascular disease and risk factors, AF duration, risk scales, and AF subtypes (with paroxysmal AF as the reference category), was used to evaluate the independent predictors of OAC, VKA, DOAC, and antiplatelets’ therapy. Results were expressed as odds ratio (ORs) and 95% confidence intervals (CI). All P values are based on a two-sided test and a significance level of < 0.05. Analyses were performed using International Business Machines (IBM)-Statistical Package for the Social Sciences, Version 27.0 (Armonk, New York: IBM Corp.).

## Results

Out of the 6016 individuals of the original sample, 235 were considered non-eligible according to exclusion criteria. Among the remaining 5781 eligible individuals, the overall participation rate was 78.3%, which left 4528 participants (mean age 74.5 ± 6.8 years, 47.2% men). A total of 319 AF cases were identified, 178 (55.8%) men and 141 women (44.2%): 137 (43.0%) were paroxysmal, 68 (21.3%) persistent, and 114 (35.7%) permanent AF.

Table 1 reports the characteristics of the study population by AF subtype. Patients with permanent AF were significantly older compared with those with paroxysmal and persistent AF. Patients with paroxysmal AF were more likely to have a higher education level compared with other AF subtypes. Among cardiovascular conditions, the frequency of hypertension and heart failure was significantly higher in patients with permanent AF. Time from initial diagnosis was significantly longer in patients with permanent AF. Pharmacological cardioversion was significantly more frequently reported in paroxysmal and persistent AF, and electrical cardioversion in patients with persistent and permanent AF. Categorical distribution of CHA_2_DS_2_-VASc and HAS-BLED scores was not significantly different among AF subtypes.

**Table 1:** Distribution of baseline variables by atrial fibrillation subtypes

Variables	Paroxysmal *N* = 137	Persistent *N* = 68	Permanent *N* = 114	*P*-value	Total *N* = 319
Mean age ± SD (years)	77.8 ± 7.3	76.2 ± 6.6	80.4 ± 6.0	< 0.001	78.3 ± 6.9
Sex (men)	54.0%	50.0%	61.4%	0.279	55.8%
Home alone	16.1%	17.6%	21.1%	0.589	18.2%
High-school level or higher	35.8%	26.5%	19.3%	0.014	27.9%
Hypertension	79.6%	69.1%	85.1%	0.036	79.3%
Previous myocardial infarction	10.2%	13.2%	10.5%	0.795	11.0%
Heart failure	7.3%	10.3%	25.4%	< 0.001	14.4%
Diabetes	21.9%	20.6%	27.2%	0.501	23.5%
Hypercholesterolemia^a^	49.6%	38.2%	38.6%	0.137	43.3%
Hypertriglyceridemia^b^	12.4%	7.4%	14.9%	0.321	12.2%
Alcohol consumption	34.3%	29.4%	27.2%	0.461	30.7%
Peripheral artery disease	9.5%	5.9%	7.9%	0.669	8.2%
Renal disease^c^	13.1%	11.8%	7.0%	0.278	10.7%
Transient ischemic attack	2.2%	4.4%	4.4%	0.565	3.4%
Previous stroke	5.1%	4.4%	5.3%	0.966	5.0%
Years from AF diagnosis ± SD	5.5 ± 5.7	5.7 ± 6.1	9.1 ± 6.3	< 0.001	6.8 ± 6.2
Pharmacological cardioversion	33.6%	41.2%	16.7%	0.001	29.2%
Electrical cardioversion	8.0%	19.1%	24.6%	0.002	16.3%
CHA_2_DS_2_-VASc score				0.824	
0–1	3.6%	4.4%	5.3%		4.4%
≥ 2	96.4%	95.6%	94.7%		95.6%
HAS-BLED score				0.434	
0–2	85.4%	89.7%	90.4%		88.1%
≥ 3	14.6%	10.3%	9.6%		11.9%

*AF* atrial fibrillation, *SD* standard deviation, *CHA*_*2*_*DS*_*2*_*-VASc* congestive heart failure, hypertension, age ≥ 75 (doubled), diabetes, prior stroke or transient ischemic attack (doubled), vascular disease, age 65–74, female sex, *HAS-BLED* hypertension, abnormal renal/liver function, stroke, bleeding history or predisposition, labile international normalized ratio, elderly (> 65 years), drugs/alcohol concomitantly

^a^Total cholesterol level ≥ 200 mg/dL (5.18 mmol/L)

^b^Triglycerides level ≥ 150 mg/dL (1.7 mmol/L)

^c^Chronic dialysis, renal transplantation, or serum creatinine ≥ 2.26 mg/dL (200 μmol/L)

Table 2 reports the frequency of anticoagulant and antiplatelet therapy by AF subtypes. Overall, 69.3% of AF patients received anticoagulant therapy. The frequency of anticoagulant therapy (VKA or DOAC) was 43.0% in patients with paroxysmal AF, 85.3% in those with persistent AF, and 91.2% in patients with permanent AF (*P* < 0.001). Differences were confirmed also considering VKA or DOAC therapy separately (*P* < 0.001) and, among DOAC, mainly for dabigatran. Conversely, the frequency of antiplatelet therapy, with or without anticoagulants, was significantly higher in paroxysmal compared with permanent and persistent AF (*P* < 0.001). Patients with paroxysmal AF were also more likely to receive no antithrombotic treatment (*P* < 0.001). Differences in the frequency of anticoagulant therapy were confirmed also when the distribution by risk scale category was considered (CHA_2_DS_2_-VASc score ≥ 2 and HAS-BLED score 0–2).

**Table 2:** Oral anticoagulants and antiplatelet therapy by atrial fibrillation subtypes, and oral anticoagulants by risk scales and atrial fibrillation subtypes

	Paroxysmal *N* = 137	Persistent *N* = 68	Permanent *N* = 114	*P*-value	Total *N* = 319
*Therapy*					
OAC (VKA or DOAC)	43.0%	85.3%	91.2%	< 0.001	69.3%
With antiplatelet drugs	10.2%	11.8%	8.7%	0.806	10.0%
Without antiplatelet drugs	32.8%	73.5%	82.5%	< 0.001	59.3%
VKA	20.4%	30.9%	59.6%	< 0.001	36.7%
DOAC	22.6%	54.4%	31.6%	< 0.001	32.6%
Dabigatran	8.0%	25.0%	14.9%	0.004	14.1%
Rivaroxaban	8.8%	17.6%	9.7%	0.136	11.0%
Apixaban	5.8%	11.8%	7.0%	0.308	7.5%
Antiplatelet drugs	50.4%	22.1%	14.0%	< 0.001	31.3%
Without anticoagulants	40.2%	10.3%	5.3%	< 0.001	21.3%
No OAC or antiplatelets drugs	16.8%	4.4%	3.5%	< 0.001	9.4%
*OAC (VKA or DOAC) by risk scales*					
CHA_2_DS_2_-VASc score 0–1	60.0%	100.0%	83.3%	0.382	78.6%
CHA_2_DS_2_-VASc score ≥ 2	42.4%	84.6%	91.7%	< 0.001	68.9%
HAS-BLED score 0–2	45.3%	88.5%	93.2%	< 0.001	72.2%
HAS-BLED score ≥ 3	30.0%	57.1%	72.7%	0.063	47.4%

*OAC* oral anticoagulants, *VKA* vitamin K antagonists, *DOAC* direct-acting oral anticoagulants, *CHA*_*2*_*DS*_*2*_*-VASc* congestive heart failure, hypertension, age ≥ 75 (doubled), diabetes, prior stroke or transient ischemic attack (doubled), vascular disease, age 65–74, female sex; HAS-BLED, hypertension, abnormal renal/liver function, stroke, bleeding history or predisposition, labile international normalized ratio, elderly (> 65 years), drugs/alcohol concomitantly

**Fig. 1:**
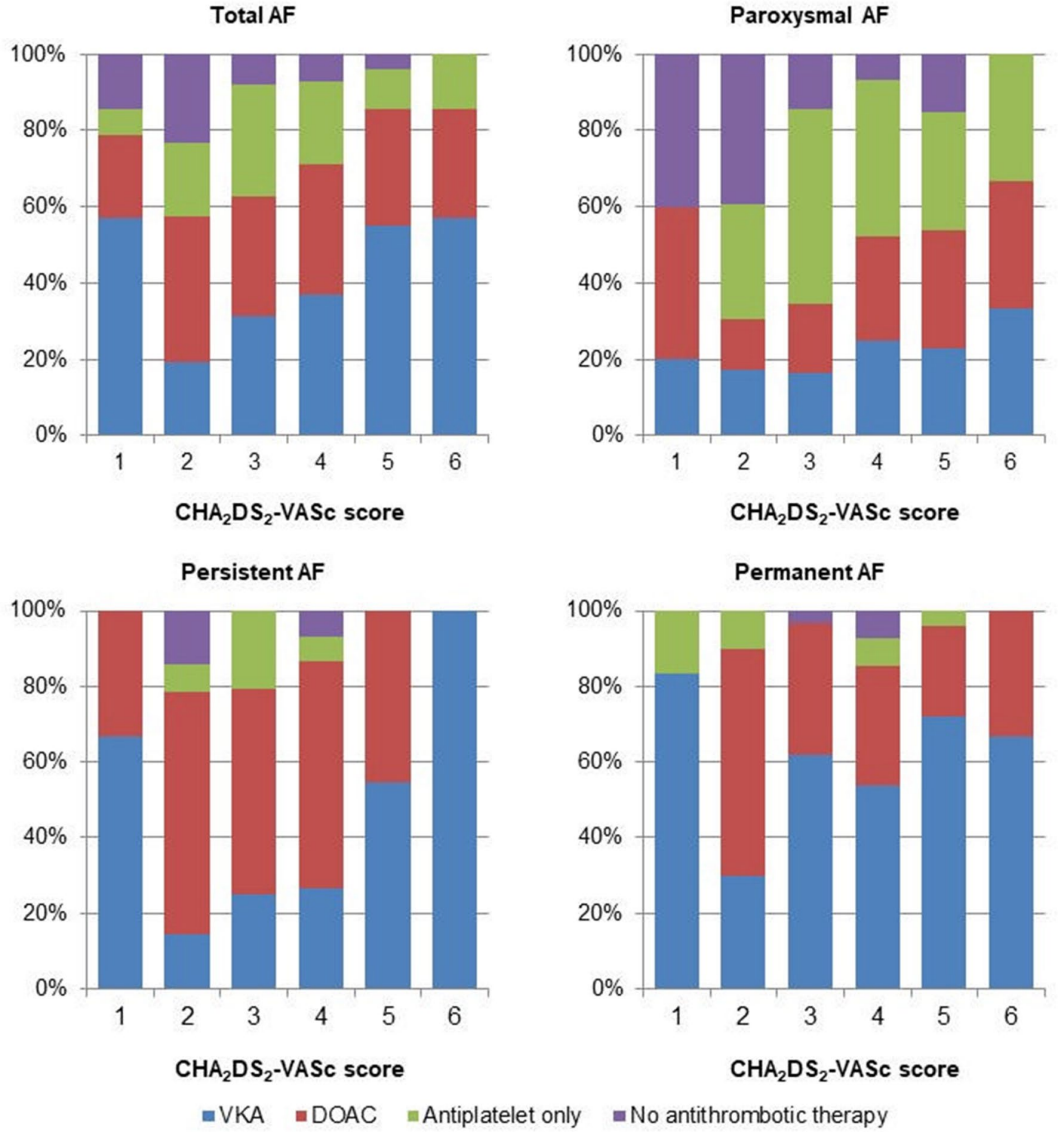
Frequency (%) of antithrombotic therapy by CHA_2_DS_2_-VASc score in total sample and by atrial fibrillation subtypes

**Fig. 2:**
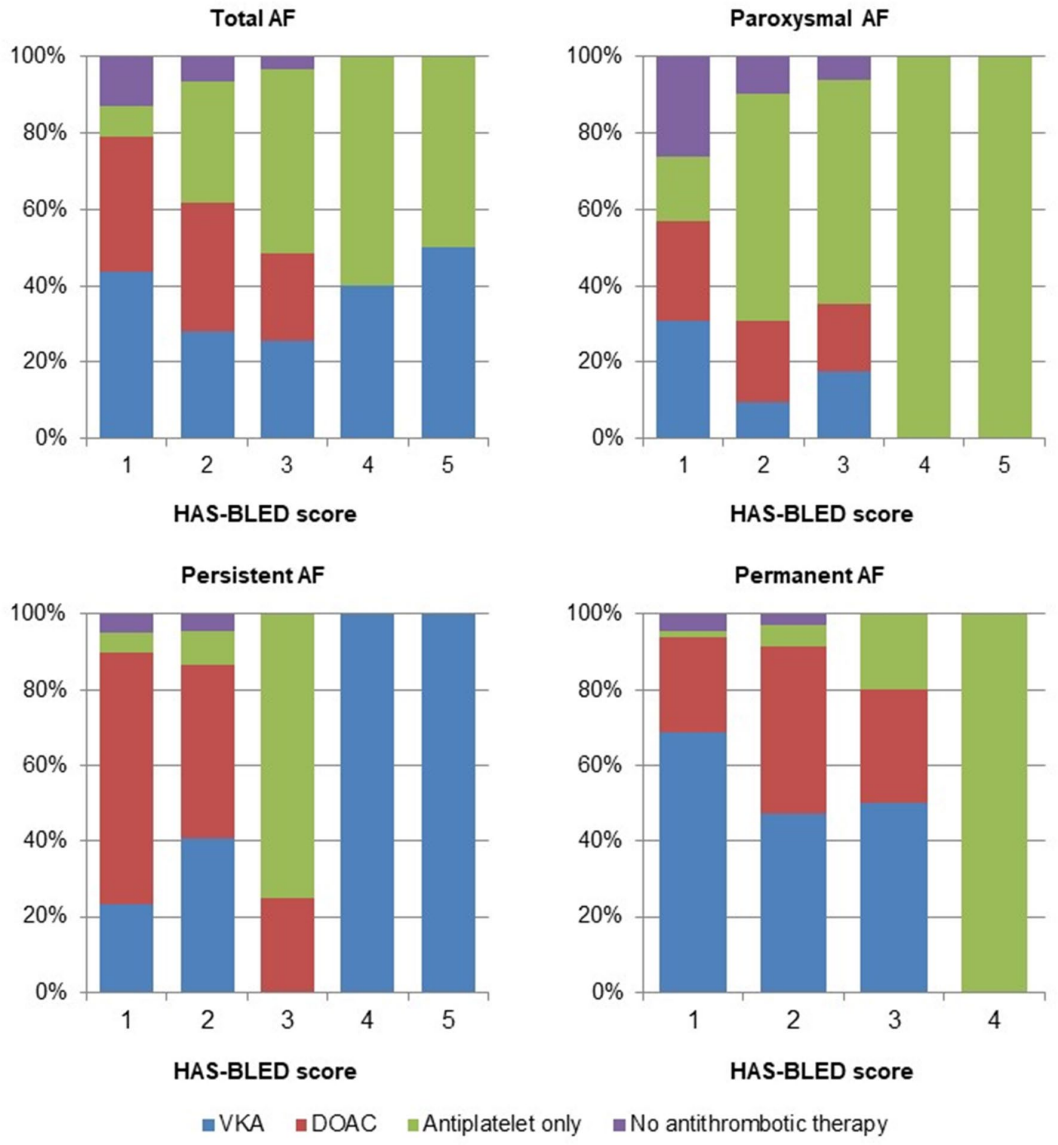
Frequency (%) of antithrombotic therapy by HAS-BLED score in total sample and by atrial fibrillation subtypes

Figure 1 shows the distribution of antithrombotic therapy by CHA_2_DS_2_-VASc score. Considering total AF, the frequency of anticoagulant therapy increased with CHA_2_DS_2_-VASc score. This trend was confirmed for all AF subtypes, but percentages were lower for paroxysmal AF, compared with both persistent and permanent forms, for all CHA_2_DS_2_-VASc scores. Frequency of treatment with antiplatelets alone decreased with increasing CHA_2_DS_2_-VASc scores, except for paroxysmal AF patients. Figure 2 shows that, in total AF, increasing HAS-BLED score was associated with a lower frequency of anticoagulant therapy, and a higher frequency of antiplatelet therapy alone. This was particularly evident for paroxysmal AF and permanent AF, where no patient with HAS-BLED score > 3 received OAC.

The independent effect of baseline variables on the probability to receive anticoagulant or antiplatelet therapy was evaluated by logistic regression analysis (Table 3). In all models, paroxysmal AF was the reference category. Having persistent (OR, 7.78; 95% CI, 3.61–16.78) or permanent (OR, 12.44; 95% CI, 5.87–26.33) AF was associated with a significant increase in the likelihood of receiving anticoagulant therapy, as well as the presence of heart failure (OR, 3.04; 95% CI,1.03–8.96), while having HAS-BLED score ≥ 3 was associated with a significant reduction of the odds of having OAC (OR, 0.31; 95% CI, 0.13–0.70).

**Table 3:** Models of logistic regression analysis for demographics, vascular disease and risk factors, AF duration, risk scales, and AF subtypes (with paroxysmal AF as the reference category) as predictors of anticoagulant or antiplatelet therapy

Variable	*P-value*	OR (95% CI)
*Model 1: OAC (VKA or DOAC)*		
AF subtype	< 0.001	
Persistent AF (ref. paroxysmal)	< 0.001	7.78 (3.61–16.78)
Permanent AF (ref. paroxysmal)	< 0.001	12.44 (5.87–26.33)
Heart failure	0.044	3.04 (1.03–8.96)
HAS-BLED ≥ 3	0.005	0.31 (0.13–0.70)
*Model 2: VKA*		
AF subtype	< 0.001	
Persistent AF (ref. paroxysmal)	0.226	1.53 (0.77–3.06)
Permanent AF (ref. paroxysmal)	< 0.001	4.37 (2.43–7.85)
Heart Failure	< 0.001	4.84 (2.28–10.29)
Electrical cardioversion	0.013	2.36 (1.20–4.65)
*Model 3: DOAC*		
AF subtype	< 0.001	
Persistent AF (ref. paroxysmal)	< 0.001	4.33 (2.30–8.15)
Permanent AF (ref. paroxysmal)	0.028	1.92 (1.07–3.42)
Heart Failure	0.005	0.29 (0.12–0.68)
*Model 4: Antiplatelets*		
AF subtype	< 0.001	
Persistent AF (ref. paroxysmal)	< 0.001	0.27 (0.14–0.54)
Permanent AF (ref. paroxysmal)	< 0.001	0.15 (0.08–0.29)
HAS-BLED ≥ 3	< 0.001	5.75 (2.62–12.64)
*Model 5: Antiplatelets without anticoagulants*		
AF subtype	< 0.001	
Persistent AF (ref. paroxysmal)	< 0.001	0.16 (0.07–0.40)
Permanent AF (ref. paroxysmal)	< 0.001	0.08 (0.03–0.19)
HAS-BLED ≥ 3	< 0.001	5.44 (2.38–12.42)

*OR* odds ratio, *CI* confidence interval, *AF* atrial fibrillation, *OAC* oral anticoagulants, *VKA* vitamin K antagonists, *DOAC* direct-acting oral anticoagulants, *HAS-BLED* hypertension, abnormal renal/liver function, stroke, bleeding history or predisposition, labile international normalized ratio, elderly (> 65 years), drugs/alcohol concomitantly

The role of AF subtypes on the probability of receiving anticoagulant therapy was confirmed also in logistic regression analyses considering separately VKA or DOAC therapy, while the positive association of heart failure was confirmed only for VKA therapy, with a negative association found between heart failure and use of DOAC. Having performed electrical cardioversion significantly increased the probability of receiving VKA therapy. Finally, the probability of antiplatelet treatment (overall or without anticoagulant treatment) was significantly higher in paroxysmal AF and in patients with HAS-BLED score ≥ 3.

## Discussion

We evaluated the possible role of AF subtypes on the use of OAC therapy in a large representative sample of the Italian older population. Compared with patients diagnosed with persistent or permanent AF, patients with paroxysmal AF were significantly less often prescribed OAC, VKA, or DOAC therapy and significantly more often given antiplatelets, with or without OAC. Patients with paroxysmal AF were also more likely to be untreated with any antithrombotic therapy. Differences were confirmed considering the distribution of CHA_2_DS_2_-VASc and HAS-BLED risk scales and in multivariate analyses including baseline variables and risk scales. HAS-BLED score ≥ 3 increased significantly the probability of antiplatelet treatment, while the frequency of treatment with antiplatelets alone decreased with increasing CHA_2_DS_2_-VASc scores, except for paroxysmal AF patients. These findings were also independent of the duration of the disease.

Our population included only patients aged 65 years and older, and the great majority was in the high-risk group for thromboembolic events. While the overall percentage of AF patients treated with OAC (69.3%) can be considered quite satisfactory, in the high-risk group, figures were 42.4% for paroxysmal, 84.6% for persistent, and 91.7% for permanent AF (*P* < 0.001). Values were similar considering the low haemorrhagic risk group according to the HAS-BLED score (*P* < 0.001). For all the three subtypes, prescription of OAC decreased from low to high haemorrhagic risk.

A possible role of AF subtypes on the use of OAC therapy was anecdotally reported in a few previous studies, but it was scarcely assessed in a systematic way. A previous analysis was performed for the period 2008–2012 in the PINNACLE Registry of the American College of Cardiology [21]. Persistent and permanent AF subtypes were combined. Patients with paroxysmal AF were significantly less frequently prescribed OAC therapy than those with persistent AF (50.4% vs. 64.3%), more frequently prescribed only antiplatelet therapy (35.1% vs. 25.0%), or neither antiplatelet nor anticoagulant therapy (14.5% vs. 10.8%; *P* < 0.001 for all three comparisons). In our survey, frequency of OAC therapy was over 85% in persistent and permanent AF, indicating a better adherence to guidelines for those subtypes, while frequency for paroxysmal AF remained low, with a percentage of 43%; the frequency of patients with paroxysmal AF given only antiplatelet therapy was similar (40.2%), while it was lower in our patients for persistent and permanent AF (10.3% and 5.3%, respectively); we had also similar frequency of patients receiving neither antiplatelet nor anticoagulant therapy for paroxysmal AF (16.8%), but lower when referring to persistent and permanent AF (4.4% and 3.5%, respectively).

In the EURObservational Research Programme-Atrial Fibrillation (EORP-AF) [22], VKA were given to 62.3% of patients with paroxysmal, 69.8% of patients with persistent, and 77.2% of those with permanent AF (*P* < 0.001). Differences were not significant for DOAC, but percentages of patients given DOAC were low: 15.2%, 13.1%, and 10.9%, respectively (*P* = 0.204).

Similarly, in the Fushimi AF Registry [23], patients with paroxysmal AF were significantly less frequently prescribed OAC therapy (40.8%) than those with persistent (56.9%) or permanent AF (67.7%) (*P* < 0.001), and in the Systematic Assessment of Geriatric Elements in Atrial Fibrillation (SAGE-AF) [24] prospective cohort study, compared with paroxysmal AF, having persistent or long-standing persistent AF significantly increased the odds or being treated with OAC at multivariate analysis (OR, 5.71; 95% CI, 3.02–10.82; and OR, 4.72; 95% CI, 1.58–14.24, respectively).

Taken together, these data indicate paroxysmal AF as an independent predictor of a reduced likelihood of anticoagulant prescription, even though the current guidelines recommend OAC treatment based on the clinical risk profile for stroke, regardless of AF type or duration [7], and recent trials clearly showed the benefits of anticoagulant therapy in patients with moderate-to-high clinical risk of stroke independently of AF pattern [13–16].

These findings could be due to the wrongfully perceived lower thrombotic risk associated with paroxysmal AF, and an intuitive belief that less AF means a lower risk of stroke [21, 25]. Therefore, treating physicians may find it easier and less risky to prescribe antiplatelet therapy to patients who are predominantly in sinus rhythm despite a clear benefit of OAC over antiplatelet therapy. Conversely, since AF is more likely to be captured by ECG in persistent/permanent AF, a better adherence to guideline-based recommendations could be explained by “seeing is believing” [21].

Heart failure was significantly associated with increased probability of receiving OAC at multivariate analysis. AF may be both cause and consequence of heart failure [26]. Recent achievements indicate a significant effect on survival in patients with AF and concomitant heart failure, with a 40% increase in mortality compared to patients in sinus rhythm [27], particularly when ejection fraction is preserved [28]. Our data suggest that treating physicians recognize the high risk of thromboembolic complications in AF patients with heart failure, and the relevance of OAC treatment in those patients independently of AF pattern. In our study, VKA seem the preferred option.

Our findings come from a population-based, multicentre study with a nationwide representative sample, which makes results transferable to the Italian older population. Data on frequency of AF subtypes were achieved through a combined screening procedure, which reduced the chance of missing AF cases, and on the direct clinical and ECG evaluation of all positive participants [29]. All the remaining clinical information was collected from the computerized practice records, and directly from the GPs and the patients.

Our study presents some limitations. First, despite the accurate screening and diagnostic procedure, we cannot exclude that some AF cases may have not been detected. Second, although the classification of AF in different subtypes was made after a 6-month observation, by an adjudication panel using all available medical information, follow-up at a later stage or further information emerging might require different treatment decisions or suggest a re-classification. In addition, a renouncing attitude towards rhythm control interventions in older patients may sometimes transform persistent into permanent form of the arrhythmia. Third, as we did not investigate time in therapeutic range in patients taking VKA, or DOAC adherence, we have no information regarding quality of OAC therapy by AF subtype, or the possible effect of adherence to treatments on outcome according to different subtypes. Fourth, considering our exclusion criteria, it was not possible to measure the impact of relevant geriatric conditions, such as dementia, on the use of anticoagulant therapy. Finally, due to evolving therapeutic opportunities, results might have been different if the analysis had been conducted in a population enrolled more recently.

## Conclusion

In our survey, involving a national sample representative of the Italian older population, patients with paroxysmal AF were significantly less often prescribed OAC, VKA, or DOAC therapy and significantly more often given antiplatelets therapy, compared with patients diagnosed with persistent or permanent AF. This was independent of clinical risk or disease duration. Increased adherence to available evidence and guidelines recommendations is essential to reduce the future burden of this highly impacting cardiac arrhythmia.

## Data Availability

The data that support the findings of this study are property of the Italian Ministry of Health. Restrictions apply to the availability of these data, which were used under license for this study. Data are available from the authors with the permission of Italian Ministry of Health.
